# Feeling seen matters: how organization-based self-esteem mediates the relationship between university students’ coping resources and thriving in Germany, Indonesia, and the United Arab Emirates

**DOI:** 10.3389/fpsyg.2025.1527121

**Published:** 2025-09-10

**Authors:** Jannika Haase, Maila D. H. Rahiem, Madiha Hashmi, Heejung S. Kim, Lysann Zander

**Affiliations:** ^1^Division of Empirical Educational Research, Institute of Education, Leibniz Universität Hannover, Hanover, Germany; ^2^Department of Early Childhood Education, Faculty of Education, Syarif Hidayatullah State Islamic University Jakarta, South Tangerang, Indonesia; ^3^Oral Rhetoric (Public Speaking), School of Arts & Sciences, Humanities and Social Sciences Division, American University in Dubai, Dubai, United Arab Emirates; ^4^Department of Psychological and Brain Sciences, University of California, Santa Barbara, CA, United States

**Keywords:** thriving, higher education, coping resources, academic self-efficacy (ASE), social belonging (SB), organization-based self-esteem (OBSE), gender

## Abstract

**Introduction:**

While there is substantial evidence on the negative repercussions of study-related stressors on university students’ mental health and well-being, comparably less is known about a specific adaptive response to stressors in higher education: students’ thriving, that is, the experience of vitality and learning under challenging circumstances. Given the lack of comparative research on students’ adaptive outcomes in diverse cultural contexts, we examined coping resources (i.e., academic self-efficacy, ASE; social belonging, SB) as predictors of female and male students’ thriving in an individualistic culture (i.e., Germany, *n* = 259), and compared it to two collectivistic cultures (i.e., Indonesia, *n* = 839; United Arab Emirates, UAE, *n* = 230). We further investigated the role of organization-based self-esteem (OBSE) as a potential mediator between students’ coping resources and thriving.

**Methods and Results:**

Multiple-group moderated mediation analyses showed that OBSE served as a mediator between SB and thriving in all three countries, irrespective of students’ gender. ASE directly catalyzed thriving among female and male students in Indonesia, only among female students in the UAE, but not in Germany. SB directly contributed to female and male students’ thriving in Germany and Indonesia.

**Discussion:**

Our findings point to the universal decisive role of OBSE in enabling students in different cultures to transform coping resources into experiences of thriving when facing study-related stressors.

## 1 Introduction

During their studies, university students face various study-related stressors such as managing a high workload, studying for and taking exams, but also establishing relationships in a new social environment (Abouserie, 1994; Pluut et al., 2015; Yang et al., 2021). While students in all cultures face study-related stressors (Aristovnik et al., 2020), the perception of and responses to these stressors can vary greatly (Marques-Pinto et al., 2025; Mohamed et al., 2022). Established models on stress, coping and resilience posit that people, when facing stressors, can show negative or positive responses (Transactional Model of Stress and Coping, TSC, Lazarus and Folkman, 1984, 1987; see also Carver, 1998; O’Leary and Ickovics, 1995; Park, 1998). According to these theories, some individuals respond negatively, for example with increased levels of stress experiences (Lazarus and Folkman, 1984), while others can show adaptive responses (Leipold and Greve, 2009), even a sense of stress-related growth (Park et al., 1996). One form of stress-related growth is thriving, i.e., a positive psychological state characterized by a joint sense of vitality (affective) and learning experiences (cognitive; Ozcan et al., 2023; Porath et al., 2012; Spreitzer et al., 2005). Prior research found that individual (e.g., self-efficacy) and social (e.g., social support) predictors facilitate experiences of thriving (e.g., Feeney and Collins, 2015; Imran et al., 2020; Kim and Beehr, 2020; Kleine et al., 2019; see also Brown et al., 2017; Liu et al., 2021). These findings have been replicated in Western and non-Western contexts for both individual and social predictors (e.g., Christensen-Salem et al., 2021; Jiang et al., 2020a; Kleine et al., 2019; Yang et al., 2023).

Students’ thriving can be considered a positive outcome of their coping process when experiencing study-related stressors as challenges (see TSC, Lazarus and Folkman, 1984, 1987; O’Leary et al., 1998; Park, 1998). Central to the current study is the prediction that, when facing study-related stressors, students’ academic self-efficacy (ASE, i.e., beliefs about their ability to perform academic tasks at predefined levels; Höhne and Zander, 2019; Jerusalem and Schwarzer, 1986; Travis et al., 2020)**—**as an indicator of available individual coping resources**—**and social belonging to the study program (SB, i.e., the extent to which students perceive themselves as part of their study program, Kreutzmann et al., 2018; Murphy et al., 2020)**—**as an indicator of available social coping resources**—**predict students’ thriving. To be able to fully transform the beneficial effects of these available resources into thriving, students should feel socially validated, i.e., feel valued, seen and important in their study program. To test this proposition, we introduced and included a construct developed and widely established in work and organizational contexts and adapted it for higher education: students’ organization-based self-esteem (OBSE; Pierce et al., 1989; for a recent study see Zhou et al., 2023b). Building on research that conceptualizes self-esteem as a hierarchical construct varying across contexts, with OBSE as a context-specific sub-dimension of general self-esteem (Bowling et al., 2010; Kim et al., 2024; Korman, 1976; Pierce and Gardner, 2004; Pierce et al., 1989; Rosenberg et al., 1995; see also Kim et al., 2024), and recognizing OBSE as a key predictor of psychological outcomes such as thriving at work (e.g., Kim and Beehr, 2020), we considered it critical to investigate the role of OBSE in fostering thriving within higher education settings. To date, as far as we know, no cross-cultural study has investigated the role of OBSE as a mediator within the same analysis in higher education. Given that models of thriving have predominantly been developed using WEIRD samples (Western, Educated, Industrialized, Rich, Democratic; Henrich et al., 2010; Muthukrishna et al., 2020; Porath et al., 2012), we sought to examine the generalizability of these models by comparing the role of key predictors of thriving across diverse cultural contexts. Specifically, we investigated coping resources (i.e., ASE, SB) as predictors of students’ thriving, potentially mediated by OBSE. We conducted cross-cultural comparisons between a German sample and two culturally distinct samples: one from Indonesia and one from the United Arab Emirates (UAE). These countries were selected based on their contrasting positions on two key cultural dimensions**—**individualism and power distance (Hofstede, 1980, 2001, 2011)**—**as well as documented differences in academic cultures (Hofstede, 1986; Irnidayanti and Fadhilah, 2023; McMinn et al., 2022; see section 2.1). Moreover, since experiences of thriving can depend on gender role beliefs (e.g., Di Milia and Jiang, 2024; Feeney and Collins, 2015), which, in turn, are contingent on the cultural context (e.g., Cuddy et al., 2015; Kosakowska-Berezecka et al., 2023), we examined gender as a potential moderator. In the present research, we therefore consider a novel potential interaction of two moderators in research on thriving in higher education: culture and gender.

## 2 Theoretical framework

### 2.1 Understanding students’ thriving in different cultures

The concept of thriving has gained popularity among scholars in organizational psychological research (e.g., Goh et al., 2023; Jiang et al., 2020b; Porath et al., 2022; Shahid et al., 2021). Although there is notable diversity in the theoretical and methodological conceptualization of thriving (for an overview see Brown et al., 2017), there is some agreement on the idea that experiences of thriving are characterized by two main dimensions: a sense of both vitality (affective) and learning (cognitive; Porath et al., 2012; Spreitzer et al., 2005; for a current example see Warnock et al., 2024). People who thrive at work experience personal growth by feeling energized and alive, and by constantly acquiring and applying knowledge as well as by continually improving and getting better at what they do (Chen et al., 2021; Porath et al., 2012). Experiencing thriving, thus, goes beyond merely managing or handling adverse situations (Chen et al., 2021; Feeney and Collins, 2015). Individuals can thrive when they feel they are growing as a result of the adversity and challenge they encountered; it is more than restoring the status quo (Feeney and Collins, 2015; Sun et al., 2022). In line with that, thriving has the potential to contribute to positive work-related outcomes such as health (e.g., Warnock et al., 2024), job satisfaction (e.g., Jiang et al., 2020a), and career intentions as well as performance (e.g., Christensen-Salem et al., 2021; Ozcan et al., 2023). So far, research on thriving has been primarily conducted in work and organizational contexts with adults, across a variety of work domains (e.g., Basinska and Rozkwitalska, 2022; Jiang et al., 2020a; Paterson et al., 2014; Walumbwa et al., 2018; Yang et al., 2023; for an overview see Kleine et al., 2019), but remains scarce in higher education (e.g., Ozcan et al., 2023; Sahin and Tuna, 2022).

Both the process of coping with and psychological outcomes from challenging situations may vary depending on the cultural context in which these factors are assessed (Kluckhohn and Strodtbeck, 1961; Ngamaba and Soni, 2018; Tang et al., 2021; Ye et al., 2015). Although multiple theoretical frameworks offer valuable lenses for examining cultural variation (e.g., Hofstede, 1980, 2001, 2011; Inglehart and Baker, 2000; Schwartz, 1999), we selected Hofstede’s cultural dimensions (Hofstede, 1980, 2001, 2011; Hofstede and Bond, 1984) given their foundation in organizational psychology, which aligns with the conceptual roots of the thriving at work construct (Spreitzer et al., 2005). Thriving, along with its two primary predictors examined in this study, reflects psychological processes that are likely to be shaped by cultural context (e.g., Fatehi et al., 2020; Jin et al., 2023; Kitayama and Salvador, 2024). To address this cultural variability, we focused on two of Hofstede’s dimensions—individualism and power distance—as key cultural factors likely to influence these processes (eg., Fatehi et al., 2020; Jin et al., 2023; Kitayama and Salvador, 2024). In individualistic cultures, people experience themselves as separate entities whose self-cognition refers to themselves as (emotionally) independent, unique and distinct units from others, focusing on autonomy and individual goals (Hofstede and McCrae, 2004; Kitayama and Salvador, 2024; Markus and Kitayama, 1991, 2010). Social relationships are often considered sources that can verify and affirm the individual self (Markus and Kitayama, 1991, 2010). By contrast, in collectivistic cultures, people consider themselves an extension of the various social systems they belong to, experiencing their identity as located in their group memberships (Markus and Kitayama, 1991, 2010). Their self-cognition refers to themselves as rather socially dependent, focusing on group goals, interpersonal duties and harmonious social relationships (Hofstede and McCrae, 2004; Kitayama and Salvador, 2024; Markus and Kitayama, 1991, 2010; see also Kitayama et al., 2000; Kitayama and Uskul, 2011; Triandis et al., 1990; for the complexity of the dimensions individualism–collectivism and independence-interdependence see Lomas and Diego-Rosell, 2023). Because we examined university students within their respective academic institutions, we considered the variation in academic cultures across the cultural contexts examined in this study, which, in turn, is related to the cultural dimension power distance (Hofstede, 1986, 2001). Hofstede (1986, 2001) argued that in low power distance cultures, i.e., cultures where less powerful members of organizations are less likely to expect and less willing to accept unequal power distribution, teaching is frequently learner-centered and instructors are expected to treat students more as equals. By contrast, in high power distance cultures, i.e., cultures where less powerful members of organizations expect and accept that power is distributed unequally, instruction in educational institutions is typically instructor-centered and strictly disciplined. As a consequence, group work and student interaction are often more fostered in low power distance cultures than in high power distance cultures (e.g., Frambach et al., 2014; Fry and Bi, 2013; Hofstede, 2001; Irnidayanti and Fadhilah, 2023; Kelo and Iucu, 2025; King, 2020; for the complex relationships between student-centered learning and culture see also Ping et al., 2024). According to Hofstede’s cultural dimensions scores (Hofstede, 1980; The Culture Factor Group, 2025), Germany is considered an individualistic culture with a high individualism index score (individualism = 79). Further, it has a low power distance score (power distance = 35; Hofstede, 1980; The Culture Factor Group, 2025). In line with Hofstede (1986, 2001), German universities tend to adopt a more learner-centered approach, incorporating various group-based learning arrangements (BMBF, 2020). Indonesia is considered a collectivistic culture with a low individualism index score (individualism = 5; Hofstede, 1980; The Culture Factor Group, 2025). Moreover, it has a high power distance score (power distance = 78; Hofstede, 1986; The Culture Factor Group, 2025). In Indonesia, higher education is predominantly instructor-centered with a significant amount of frontal teaching (Hofstede, 1986; Irnidayanti and Fadhilah, 2023). The UAE is also considered a collectivistic culture with a low individualism index score (individualism = 36) and a high power distance culture with a high power distance score (power distance = 74; Hofstede, 1980; The Culture Factor Group, 2025). However, when compared to Indonesia, higher education instruction in the UAE is strongly oriented toward academic cultures in low power distance cultures, mostly also adopting a learner-centered approach (e.g., McMinn et al., 2022). Unfortunately, both cultural dimensions and academic cultures have not been assessed in our study.

Since measurement invariance could not be established between Indonesia and the UAE (see section 3.1), limiting full comparability, Germany served as a reference point in our study. While Germany differs significantly from Indonesia in both cultural dimensions and academic cultures, we initially included the UAE to introduce a theoretically meaningful contrast—combining cultural differences with Germany and academic-related differences with Indonesia. This tension between broader cultural dimensions and institutionalized educational practices highlights the UAE as a theoretically relevant case for examining whether the psychological mechanisms under study may function similarly or differently across contexts that differ—to varying degrees—not only in cultural dimensions but also in academic cultures (e.g., Kitayama and Salvador, 2024). We further took into account a relevant aspect differentiating the examined countries in our study, that is, the varying levels of gender inequality with regard to higher social and economic status of men compared to women (World Economic Forum, 2024), being aware that this could confound effects attributed to cultural differences with regard to the cultural dimensions proposed by Hofstede (1986, 2001, 2011). Gender inequality remains notably more pronounced in Indonesia and the UAE compared to Germany, where it is comparatively very low (World Economic Forum, 2024). Notably, gender inequality has been found to be perceived as more unfair by women in individualistic cultures than in collectivistic cultures (e.g., Kinias and Kim, 2012) and to be negatively related to well-being among women from liberal countries (i.e., high in individualism), but not among those from conservative countries (i.e., high in collectivism; Li et al., 2025).

### 2.2 Individual and social predictors of thriving in different cultures

Bandura (2002) proposed that believing in one’s efficacy to produce desired effects under challenging conditions would be cross-culturally relevant. Accordingly, a study with 19,120 participants from 25 countries, including Germany and Indonesia (Scholz et al., 2002), found that despite different mean levels of self-efficacy across cultures, general self-efficacy was structurally equivalent across cultures (for a recent study see Gebauer et al., 2021). However, it should be noted that extensive research highlights the significance of cultural values shaping self-efficacy, confirming that self-efficacy is related to variations of self in its fundamental composition (e.g., Jin et al., 2023; Kitayama and Uskul, 2011; Klassen, 2004; Liu et al., 2022; see also Bonneville-Roussy et al., 2019). For instance, higher levels of self-efficacy among students in individualistic cultures compared to those in collectivistic cultures (e.g., Jin et al., 2023) are often explained by culturally specific foundations of self-efficacy. In individualistic cultures, it tends to be more rooted in information about personal accomplishments, whereas in collectivistic cultures, it is more closely related to information about group success (e.g., Wang et al., 2020; see also Oettingen, 1995).

As noted, self-efficacy was found to be an individual predictor of thriving in both individualistic (e.g., Christensen-Salem et al., 2021) and collectivistic cultures (e.g., Yang et al., 2023): the more people believed they would be capable to handle challenging situations, the more they experienced vitality and learning under actually challenging circumstances. This could lead to the hypothesis that ASE would impact students’ thriving in all three examined countries. However, two alternative competing hypotheses can be considered. On the one hand, studies show that self-enhancement is more prevalent among people in individualistic cultures than in collectivistic cultures (e.g., Falk et al., 2009; Heine and Hamamura, 2007; Salvador et al., 2024). This differing valuation of the self could lead to the hypothesis that ASE may be more beneficial for students’ thriving in Germany than for students’ thriving in Indonesia and the UAE. On the other hand, different baseline levels of ASE (i.e., potentially higher values in Germany, lower values in Indonesia and the UAE; see Jin et al., 2023; Scholz et al., 2002) might suggest the hypothesis that ASE could be more beneficial in Indonesia and the UAE since having relatively lower levels of certain factors may have a stronger impact on positive outcomes such as thriving (e.g., Pavani et al., 2019).

Social relationships have been proposed and found to facilitate thriving mainly by affording individuals with the social resources to make use of their full potential to successfully handle challenges (e.g., Feeney and Collins, 2015; Jiang et al., 2020a). Building on theories on social support and attachment (Bowlby, 1973; Mikulincer and Shaver, 2007), Feeney and Collins (2015) argued that thriving through relationships happens through an interpersonal process of fortification, which includes supporting the development of others’ strengths and abilities relevant to coping with adversities. Since social relationships can signal attachment and acceptance from significant others, they can act as a secure basis for the exploration of a challenging situation. Successfully handling the situation, in turn, contributes to the experience of growth by emerging from the challenge as more vital and knowledgeable (Feeney and Collins, 2015). Social relationships have been found to predict thriving in both individualistic (e.g., Frazier and Tupper, 2018; for an indirect effect via strengths use see also Moore et al., 2022) and collectivistic cultures (e.g., Imran et al., 2020; Jiang et al., 2020a). Individuals can access social support when they experience social belonging (SB), which means that they feel included, accepted, and respected in a given environment (Walton and Brady, 2017). The need to belong is one of the most important human cross-culturally relevant needs (Allen et al., 2021; Baumeister and Leary, 1995). Students’ SB is related to adaptive socioemotional development and well-being (conceptually close to thriving) in schools and higher education in both individualistic and collectivistic cultures (e.g., Chiu et al., 2016; Cortina et al., 2017; Li et al., 2025; Suhlmann et al., 2018; Walton and Cohen, 2011). This could lead to the hypothesis that SB would predict students’ thriving in all three examined countries. However, as in the case of ASE, two other competing hypotheses could be considered. On the one hand, the greater general expectation of communion in collectivistic cultures (e.g., Fatehi et al., 2020; Sugihara and Katsurada, 2000) compared to individualistic cultures could be more strongly related to positive outcomes such as thriving in collectivistic cultures (i.e., Indonesia, UAE). This would be in line with studies suggesting that communion is a more important coping mechanism for individuals in collectivistic cultures than in individualistic cultures. For example, a recent study with Koreans and U.S. individuals has shown that there was a stronger relationship between communion and daily emotions in Korea compared to the U.S. (Joo et al., 2024). On the other hand, studies have shown that low levels of social belonging, such as social exclusion, more strongly predicted lower fulfillment of psychological needs in individualistic cultures than in collectivistic cultures (e.g., Pfundmair et al., 2015). Similarly, existential isolation was significantly related to prolonged grief symptoms in individualistic, but not in collectivistic cultures (Zhou et al., 2023a). Additionally, studies have shown that individuals’ (biological) well-being was related to supportive relationships only in individualistic cultures (Chiang et al., 2013), and that social trust contributed more strongly to well-being in individualistic than in collectivistic cultures (Guo et al., 2022). This could, in turn, lead to the assumption that SB might be more beneficial in Germany than in Indonesia and the UAE.

In general, it is important to note that people in various cultures may use varying units of reflection when assessing social factors. In individualistic cultures, assessments are often anchored in the individual self, while in collectivistic cultures they are rooted in interpersonal relationships (e.g., Fatehi et al., 2020; Markus and Kitayama, 1991).

### 2.3 The role of gender for students’ thriving

With regard to the individual coping resource in our study (i.e., ASE), so far, typically no gender differences have been found in the relationships between self-efficacy and adaptive outcomes that are conceptually close to thriving such as life satisfaction and learning engagement, in both individualistic (e.g., Robinson et al., 2022; Vecchio et al., 2007) and collectivistic cultures (e.g., Wang et al., 2022).

With regard to the social coping resource (i.e., SB), Feeney and Collins (2015) argued that experiences of thriving via social factors are contingent on gender role beliefs.^1^ Men are often stereotyped as more self-oriented, independent and oriented toward agency than women (e.g., emphasizing assertiveness of the self and separation from others). By contrast, women are often stereotyped as more other-oriented, interdependent and oriented toward communion than men (e.g., emphasizing community and creation of unions; e.g., Eagly, 1987; Eagly and Steffen, 1984; Kite et al., 2008; Kosakowska-Berezecka et al., 2023). These inferred gender-stereotypic traits have been found to be cross-culturally general in the past (e.g., Williams and Best, 1982, 1990) and in recent studies (e.g., Kosakowska-Berezecka et al., 2024).

Cuddy et al. (2015), however, have provided evidence for the cultural moderation of gender stereotypes hypothesis, according to which the stereotypic characteristics ascribed to men converge more with the characteristics that are culturally valued than the stereotypic characteristics ascribed to women. Given that independence/agency is more culturally valued in individualistic cultures than collectivistic cultures, and interdependence/communion is more culturally valued in collectivistic cultures than individualistic cultures, this could imply that SB should be important only for female, but not male students’ thriving in individualistic Germany, and male, but not female students’ thriving in collectivistic Indonesia and the UAE. Nevertheless, findings on agency/communion in different cultures have been mixed with studies showing that women were perceived or described themselves as more communal or warm than men in individualistic cultures (e.g., Bye et al., 2022; Kosakowska-Berezecka et al., 2023; Steinmetz et al., 2014), while other studies have indicated that German men rated themselves as similarly communal to German women (e.g., Obiama et al., 2022). Likewise, in collectivistic cultures, relatively more communal traits were prescribed to men compared to women (e.g., Kosakowska-Berezecka et al., 2024), but other studies have shown that women were perceived as more communal than men (e.g., Obiama et al., 2022), or that both genders were perceived as similarly communal, indicating a general expectation of communion (e.g., Steinmetz et al., 2014; Sugihara and Katsurada, 2000). This aligns with research in different cultures showing that differences between self- and other-ratings for both agency and communion of women and men—although generally large—are sometimes smaller, have been decreasing over time, and have become less traditional (e.g., Eagly et al., 2020; Hentschel et al., 2019; for a meta-analysis see Hsu et al., 2021).

### 2.4 Organization-based self-esteem (OBSE) as a potential mediator

OBSE was originally defined as the degree to which employees believe that they are valued and important within an organization (Pierce et al., 1989; see also Pierce et al., 1993). Transferring this concept to higher education, we define OBSE as the degree to which students feel seen, valued by, and important for their fellow students and their instructors, and that their opinion counts in their study program. Because self-esteem is contingent on positive evaluations of self and others as well as own accomplishments (Kwan et al., 2009), OBSE has been found to be positively related to individual (e.g., self-efficacy) and social factors (e.g., interpersonal interactions with colleagues or supervisors, organizational support; for an overview see Bowling et al., 2010; Pierce and Gardner, 2004).

To date, self-efficacy and OBSE have been found to be positively related in individualistic cultures (e.g., Bowling et al., 2010; Gardner and Pierce, 1998); however, to our knowledge, this relationship has not been investigated in collectivistic cultures. Furthermore, to our knowledge, no study has systematically examined the moderating role of gender in the relationship between self-efficacy and OBSE in higher education. Positive associations between social factors such as workplace relationships and support by colleagues, and OBSE have been found in both individualistic (e.g., Kim and Beehr, 2022) and collectivistic cultures (e.g., Su et al., 2022; Yang et al., 2018; for an overview see Bowling et al., 2010). Further, research has shown that OBSE predicts positive outcomes such as thriving, well-being, satisfaction, and engagement in both individualistic (e.g., Kim and Beehr, 2020; Pierce et al., 2016) and collectivistic (e.g., Yang et al., 2018; Zhou et al., 2023b) cultures. Additionally, OBSE has been found to be critical in challenging work conditions by mediating the effects of employees’ social (coping) resources on positive (work-related) outcomes (for individualistic cultures see Kim and Beehr, 2020, 2022; for collectivistic cultures see Gardner et al., 2018; Lee and Peccei, 2007; Yang et al., 2018; Zhiqiang et al., 2021; see also Haar and Brougham, 2016). For instance, in a study with U.S. employees, OBSE mediated the relationship between organizational feedback and employees’ thriving at work (Kim and Beehr, 2020). In China, for example, experiences of workers that support was available from their organization have been found to predict employees’ OBSE, which, in turn, predicted their family satisfaction (Yang et al., 2018). Transferring these relationships to higher education, it can be assumed that university students with high levels of OBSE, strongly derived from their positive social learning-, and performance-related experiences at university, may actively engage in tasks and strive for effective functioning and academic development, which manifest in feelings of vitality and learning (e.g., Bowling et al., 2010; Kim and Beehr, 2020; Spreitzer et al., 2005; Zhou et al., 2023b). Research conducted in individualistic cultures has consistently reported no gender effects when investigating the mediating role of OBSE between social factors and work-related outcomes such as thriving or organizational deviance (e.g., Ferris et al., 2009; Kim and Beehr, 2020; for a meta-analysis see Bowling et al., 2010). In collectivistic cultures, research has also indicated no gender effects when investigating OBSE as a mediator between social factors and work-related outcomes such as performance (e.g., Chan et al., 2013; Liu et al., 2013; Loi et al., 2020).

OBSE has been separately validated in individualistic and collectivistic cultures (e.g., Kanning and Hill, 2012; Renz et al., 2021) and examined within multinational enterprises with employees from different cultures (e.g., Vora and Kostova, 20**20**). However, to our knowledge, it has not yet been investigated within the same cross-cultural analysis in higher education.

### 2.5 The present research

To better understand the role of coping resources (i.e., academic self-efficacy, ASE; social belonging, SB) for university students’ thriving in individualistic and collectivistic cultures, as well as the potential importance of the organization-based self-esteem (OBSE) as a mediator, and to examine gender as a potential moderator of these relationships, we conducted two comparative analyses (using the same sample from Germany):

- Analysis 1: Germany and Indonesia.
- Analysis 2: Germany and the UAE.

We conducted our analyses based on the following research questions:

1. Does ASE (individual coping resource) predict thriving (Analysis 1 and Analysis 2)?
2. Does SB (social coping resource) predict thriving (Analysis 1 and Analysis 2)?
3. Does OBSE mediate these potential relationships (Analysis 1 and Analysis 2)?
4. Does gender moderate these potential direct and indirect relationships (Analysis 1 and Analysis 2)?

Based on the research discussed, with regard to research question 1, we explored whether ASE would predict students’ thriving (Analysis 1 and Analysis 2).

With respect to research question 2, we explored whether SB would contribute to students’ thriving (Analysis 1 and Analysis 2).

With regard to research question 3, we explored whether OBSE mediated the potential relationships between ASE and thriving (Analysis 1 and Analysis 2). We expected students’ SB to predict OBSE, which, in turn, would predict their thriving (Analysis 1 and Analysis 2).

Regarding research question 4, we expected the potential relationships between ASE and thriving to be irrespective of students’ gender (Analysis 1 and Analysis 2). Further, based on the mixed findings and theoretical predictions regarding gender roles, we explored the moderating role of gender in the potential relationships between SB and thriving (Analysis 1 and Analysis 2). Additionally, we explored whether the potential mediation of OBSE in the relationships between ASE and thriving would be moderated by gender (Analysis 1 and Analysis 2). We expected OBSE to mediate the relationship between SB and thriving among both female and male students in Germany, Indonesia, and the UAE (Analysis 1 and Analysis 2).

## 3 Methods

### 3.1 Participants

In Analysis 1, our sample consisted of 1,098 students (*n* = 259 from Germany; *n* = 839 from Indonesia) of a large German state university (about 30.000 enrolled students) and a medium-large state Indonesian university (about 8.000 enrolled students). Among participants who indicated their gender, 77.3% were female and 22.7% were male. Among participants who reported their degree, 69.3% were Bachelor students and 30.7% were Master students. Within the group of participants who provided information about studying in a teacher training program, 80.2% were teacher training students and 19.8% were no teacher training students. In Analysis 2, our sample consisted of 489 students (*n* = 259 from Germany: identical sample used in Analysis 1; *n* = 230 from the UAE) at the same German university and a private Emirati university (about 2.000 enrolled students). Among participants who reported their gender, 70.1% were female and 29.9% were male. Within the group of participants who indicated their degree, 77.2% were Bachelor students and 22.8% were Master students. Among participants who provided information about studying in a teacher training program, 35.3% were teacher training students and 64.7% were no teacher training students (all percentages are corrected for missing values; for the absolute frequencies, including missing values, as well as for sample characteristics separated by country see Supplementary Table 1). The majority of students at the private Emirati university were non-Emirati students, primarily from Lebanon, Egypt, Jordan, and Syria. Since measurement invariance was not supported with regard to the comparison between Indonesia and the UAE, we could not include all three countries in a single statistical analysis. However, because we aimed at confirming measurement invariance, and, thus, the cross-cultural validity for thriving and OBSE across the three countries (see Preliminary Analyses in the Supplementary material), we decided to conduct two separate sub-analyses: one comparing Germany (as an individualistic culture) with Indonesia (as a collectivistic culture), and another comparing Germany with the UAE (another collectivistic culture).

### 3.2 Procedure

Data was collected online using the web-based survey software LimeSurvey (Limesurvey GmbH, 2022). Questionnaires were distributed via a university mailing list (Germany, Indonesia) or an internal university learning portal (UAE) with an included link to the survey. In addition, in Indonesia and the UAE, questionnaires were administered during regular class hours. Students were asked to describe their study experiences in the respective semester (November 2022: winter semester 2022/23 in Germany, summer semester 2022/23 in Indonesia and the UAE). Before starting the survey, students were informed about the voluntary character of the study and the anonymity of their data. Students gave their written consent at the beginning of the survey. Students were not rewarded for their participation.

### 3.3 Measures

Before conducting the survey, measures were translated from the respective original language into German, Indonesian and English, and were then administered in German (Germany), Indonesian (Indonesia) and English (UAE). Identical scales were used in both analyses. ASE, SB, OBSE and thriving were pretested in the three countries to ensure linguistic and cultural appropriateness. After the pretesting, no further linguistic modifications to the translations were required.

#### 3.3.1 Thriving

Students’ sense of thriving was assessed using a shortened version of the well-established scale by Porath et al. (2012; original: English), representing the two dimensions vitality and learning, using a 5-point Likert response scale (1 = strongly disagree, 5 = strongly agree). The scale consisted of three items, e.g., “I feel alive and vital” (vitality) and “I am finding new ways to develop” (learning). All items showed acceptable internal consistency (Analysis 1: Cronbach’s *α* Total = 0.71, Cronbach’s α Germany = 0.72, Cronbach’s α Indonesia = 0.70; Analysis 2: Cronbach’s α Total = 0.70, Cronbach’s α Germany = 0.72, Cronbach’s α UAE = 0.68). To assess thriving—including both vitality and learning—the scale developed by Porath et al. (2012) is the most widely established and has been validated across diverse cultures (eg., Jiang et al., 2020a; Kleine et al., 2023; Lee and Lee, 2021).

#### 3.3.2 Academic self-efficacy (ASE)

Students’ beliefs about their ability to accomplish academic tasks in their study program were measured by an adapted and shortened two-item scale by Jerusalem and Schwarzer (1986; original: German) that has been used previously in the context of higher education (Höhne and Zander, 2019). While the original scale by Jerusalem and Schwarzer (1986; see also Schwarzer and Jerusalem, 1995) has also been used in higher education contexts (e.g., da Silva et al., 2020), we used the adapted version to ensure a domain-specific focus on academic self-efficacy, which can be closely related to positive psychological experiences in educational settings (e.g., Tian et al., 2024). Example items included: “I can cope with difficult situations and challenges in my studies if I try hard”; “I think that I will be able to acquire even complicated contents in courses.” Both items used a 5-point Likert response scale (1 = strongly disagree, 5 = strongly agree) and formed sufficiently reliable scales (Analysis 1: Spearman-Brown coefficient Total = 0.67, Spearman-Brown coefficient Germany = 0.69, Spearman-Brown coefficient Indonesia = 0.64; Analysis 2: Spearman-Brown coefficient Total = 0.77, Spearman-Brown coefficient Germany = 0.69, Spearman-Brown coefficient UAE = 0.85).

#### 3.3.3 Social belonging (SB)

Students’ perceived sense of social belonging to the study program was assessed using an adapted visual single-item measure by Kreutzmann et al. (2018; original: German; based on Aron et al., 1992). Students were asked: “To what extent do you perceive yourself as part of your study program?” and had to choose a graphic illustration that best symbolized how much they felt they were part of their study program using a Likert scale from 1 (“I do not feel like I belong to this study program”) to 5 (“I feel like I very much belong to this study program”). The original scale by Aron et al. (1992) has been recommended for achieving comparability across diverse contexts (e.g., Kreutzmann et al., 2018).

#### 3.3.4 Organization-based self-esteem (OBSE)

The extent to which students felt seen, valued by and important for their fellow students and their instructors, as well as to which they felt that their opinion counted in their study program, was measured using an adapted and shortened version by Kanning and Schnitker (2004; original: German) of the original scale by Pierce et al. (1989). The scale consisted of three items using a 5-point Likert response scale (1 = strongly disagree, 5 = strongly agree), and example items included: “I have the feeling that I am important for the people in my study program” and “I think that my opinion is important here.” All items were found to be internally consistent (Analysis 1: Cronbach’s α Total = 0.80, Cronbach’s α Germany = 0.81, Cronbach’s α Indonesia = 0.80; Analysis 2: Cronbach’s α Total = 0.84, Cronbach’s α Germany = 0.81, Cronbach’s α UAE = 0.87). The scale developed by Pierce et al. (1989) is one of the most widely established measure for assessing OBSE and has been applied and validated across various cultures (e.g., Kanning and Hill, 2012; Renz et al., 2021).

#### 3.3.5 Demographics

Students indicated their gender (0 = female, 1 = male), and provided information on their age, their degree and their study program at the end of the survey.

### 3.4 Statistical analyses

For both analyses, if not stated differently, we analyzed our data using Mplus version 8.5 (Muthén and Muthén, 1998), using a maximum likelihood (ML) estimator. Mplus provides a widely established and commonly used framework for estimating complex models involving multiple-group moderated mediation analyses, offering key advantages such as full information maximum likelihood (FIML) for handling missing data, as well as flexible model comparison through chi-square difference testing and global fit indices (Muthén and Muthén, 1998; e.g., Höhne et al., 2024; see below for more information). Prior to our main analyses, measurement invariance across countries was tested for our measures thriving and OBSE within the framework of multiple-group confirmatory factor analyses (MGCFA). This allowed us to test the comparability of these measures in the subsamples Germany and Indonesia (Analysis 1), and Germany and UAE (Analysis 2), which is a statistical prerequisite for meaningful group comparisons (e.g., Steenkamp and Baumgartner, 1998; see also Lacko et al., 2022). Given that measurement invariance testing within the MGCFA framework requires measures with at least three items, it was not possible to assess measurement invariance for our two-item measure of ASE and our one-item measure of SB (see Boer et al., 2018; Byrne, 1998; Milfont and Fischer, 2010). However, based on the literature on the importance of self-efficacy and social predictors such as SB for the experience of thriving (for an overview see Brown et al., 2017), we decided to include both measures into our analyses. In a next step, descriptive statistics and bivariate correlations for all variables of interest were computed. In addition, mean differences and standardized mean differences between Germany and Indonesia (0 = Germany, 1 = Indonesia; Analysis 1) and Germany and UAE (0 = Germany, 1 = UAE; Analysis 2) were calculated using simple linear regression analyses. Before conducting our main analyses, we conducted Little’s Missing-Completely-At-Random test (MCAR; Little, 1988) within SPSS’s Missing Value Analysis option (version 28.0; IBM Corp, 2021) to examine patterns of missing data in our samples.

To examine the contribution of individual and social coping resources to students’ thriving as well as the mediating role of OBSE in both analyses, moderated by gender, multiple-group moderated mediation analyses stratified by country were performed. Missing values in Mplus were estimated using FIML (Lee and Shi, 2021; Peugh and Enders, 2004; Schafer and Graham, 2002). To avoid listwise deletion of participants with missing data on independent variables, x-variables were treated as dependent variables as a result of specifying their means and variances (Hox et al., 2015). Using the GROUPING command within Mplus (Analysis 1: 0 = Germany, 1 = Indonesia; Analysis 2: 0 = Germany, 1 = UAE), we estimated the models for the country groups simultaneously. Differences in parameters between the respective both countries were tested using a chi-square difference test. Hereby, we compared an unconstrained model in which varying parameters were allowed between both respective countries to a constrained model in which parameters were set equal (Muthén and Muthén, 1998; e.g., Zander et al., 2020). All variables except the categorical variable of gender were grand-mean centered (Aguinis, 2004; Giesselmann and Schmidt-Catran, 2020). In both studies, we regressed thriving on the individual coping resource (i.e., ASE), the social coping resource (i.e., SB) as well as the interaction between gender and both coping resources, stratified by country, while controlling for gender. Moreover, we tested whether students’ OBSE would mediate the relationships between students’ coping resources, the interactions between gender and coping resources, and thriving. We conducted simple slopes tests for the significant interaction to test whether the slopes for female and male students were significantly different from 0 (Aiken and West, 1991; Liu et al., 2017). In all analyses—except when testing measurement invariance—the variables in the models were treated as manifest constructs.

## 4 Results

### 4.1 Preliminary and descriptive analyses

In Analysis 1, scalar measurement invariance was supported for both thriving and OBSE. In Analysis 2, partial scalar measurement invariance was found for thriving, while scalar measurement invariance was again supported for OBSE. Thus, the prerequisites for mean value comparisons of these variables between Germany and Indonesia (Analysis 1), and between Germany and the UAE (Analysis 2) were met (for the detailed analyses see Preliminary Analyses in the Supplementary material). In Tables 1–4, descriptive statistics for our dependent and independent variables, as well as for the mediator, and mean differences and intercorrelations among the measures are shown. To quantify multicollinearity, variance inflation factors (VIFs) were analyzed for each of the model’s predictors due to moderate to strong correlations between some of the explanatory variables as well as between the explanatory variables and the mediator. VIFs were computed within SPSS (version 28.0; IBM Corp, 2021), while running a multiple regression analysis of thriving on all independent variables and the mediator, including country and gender. In Analysis 1, with the lowest VIF-score being 1.023 and the highest being 1.208, no significant increase of the regression coefficients’ variance due to very high linear correlations between the predictors, i.e., multicollinearity, was indicated (see Table 2). This was also the case for Analysis 2 (lowest VIF-score: 1.019, highest VIF-score: 1.618; see Table 4). According to Little’s MCAR test, data points in Analysis 1 (*χ*^2^ = 21.49, df = 18, *p* = 0.255) and in Analysis 2 (*χ*^2^ = 5.27, df = 9, *p* = 0.810) were missing completely at random.

**Table 1:** Means, standard deviations, and mean comparisons by country of the dependent, the independent variables and the mediator (Analysis 1).

		Thriving	Academic self-efficacy (ASE)	Social belonging (SB)	Organization-based self-esteem (OBSE)
	*N*	M (SD)	M (SD)	M (SD)	M (SD)
Total	1,098	3.52 (0.71)	4.09 (0.65)	3.46 (1.01)	3.20 (0.74)
Germany	259	3.10 (0.89)	4.36 (0.78)	3.26 (1.03)	3.13 (0.94)
Indonesia	839	3.63 (0.60)	4.01 (0.58)	3.51 (0.99)	3.22 (0.67)
*B* (SE)		0.533 (0.06)	−0.346 (0.05)	0.249 (0.08)	0.095 (0.06)
*p*		0.000***	0.000***	0.002**	0.112

*N* = 1,098. Numbers in the bottom two rows represent the results of the simple regression analyses with country as the independent variable. Values were estimated using Mplus and full information likelihood estimation (FIML). Mean values (M) and standard deviations (SD) were rounded to two decimal places. Country: 0 = Germany, 1 = Indonesia; *p < 0.05; **p ≤ 0.01; ***p ≤ 0.001.

**Table 2:** Intercorrelations of the dependent, the independent variables and the mediator (Analysis 1).

	1	2	3	4	5	6	VIF
1. Thriving	1	0.104***	0.023**	0.105***	0.207***	0.238***	-
2. Country		1	−0.011	−0.063***	0.069***	0.022	1.104
3. Gender			1	−0.009	−0.016	0.029**	1.023
4. Academic self-efficacy (ASE)				1	0.145***	0.117***	1.174
5. Social belonging (SB)					1	0.262***	1.183
6. Org.-based self-esteem (OBSE)						1	1.208

*N* = 1,098. Values were estimated using Mplus and full information likelihood estimation (FIML). VIF = variance inflation factor of the independent variables and the mediator (variables 2–6; results were estimated using SPSS). Country: 0 = Germany, 1 = Indonesia; *p < 0.05; **p ≤ 0.01; ***p ≤ 0.001.

**Table 3:** Means, standard deviations, and mean comparisons by country of the dependent, the independent variables and the mediator (Analysis 2).

		Thriving	Academic self-efficacy (ASE)	Social belonging (SB)	Organization-based self-esteem (OBSE)
	*N*	M (SD)	M (SD)	M (SD)	M (SD)
Total	489	3.29 (0.87)	4.27 (0.81)	3.45 (1.02)	3.42 (0.97)
Germany	259	3.10 (0.89)	4.36 (0.78)	3.26 (1.03)	3.13 (0.94)
UAE	230	3.43 (0.88)	4.17 (0.83)	3.60 (0.99)	3.67 (0.94)
*B* (SE)		0.305 (0.09)	−0.184 (0.07)	0.342 (0.10)	0.542 (0.09)
*p*		0.000***	0.011*	0.000***	0.000***

*N* = 489. Numbers in the bottom two rows represent the results of the simple regression analyses with country as the independent variable. Values were estimated using Mplus and full information likelihood estimation (FIML). Mean values (M) and standard deviations (SD) were rounded to two decimal places. Country: 0 = Germany, 1 = UAE; *p < 0.05; **p ≤ 0.01; ***p ≤ 0.001.

**Table 4:** Intercorrelations of the dependent, the independent variables and the mediator (Analysis 2).

	1	2	3	4	5	6	VIF
1. Thriving	1	0.077***	0.009	0.144***	0.351***	0.440***	–
2. Country		1	0.017	−0.046*	0.086***	0.136***	1.137
3. Gender			1	−0.027	−0.007	0.028	1.019
4. Academic self-efficacy (ASE)				1	0.184***	0.190***	1.117
5. Social belonging (SB)					1	0.558***	1.489
6. Org.-based self-esteem (OBSE)						1	1.618

*N* = 489. Values were estimated using Mplus and full information likelihood estimation (FIML). VIF = variance inflation factor of the independent variables (variables 2–6; results were estimated using SPSS). Country: 0 = Germany, 1 = UAE; *p < 0.05; **p ≤ 0.01; ***p ≤ 0.001.

### 4.2 Multiple-group moderated mediation analyses

**Figure 1:**
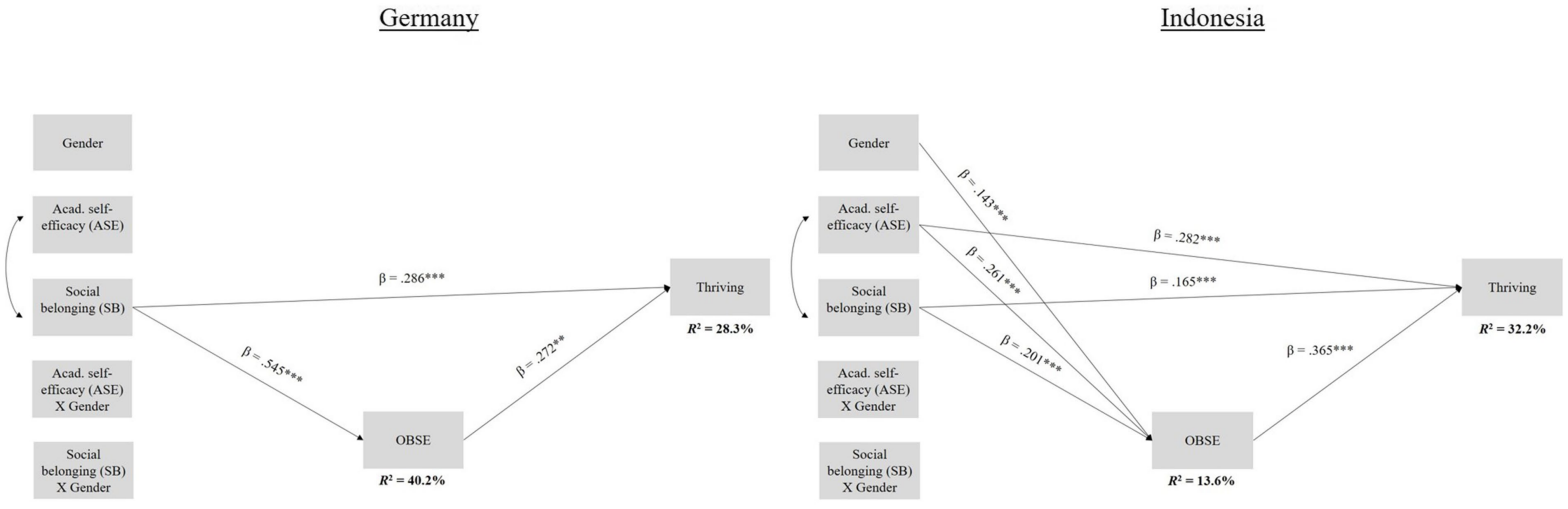
*N* = 1,098. Germany: *n* = 259; Indonesia: *n* = 839. Values were estimated using Mplus and full information likelihood estimation (FIML). 0 = Germany, 1 = Indonesia; *p < 0.05; **p ≤ 0.01; ***p ≤ 0.001. Indirect effects: Germany: partial mediation for SB (*β* = 0.149, *p* ≤ 0.01, 95% CI = 0.049 to 0.248); Indonesia: partial mediation for ASE (*β* = 0.095, *p* ≤ 0.001, 95% CI = 0.055 to 0.141), partial mediation for SB (*β* = 0.073, *p* ≤ 0.001, 95% CI = 0.041 to 0.108).

**Figure 2:**
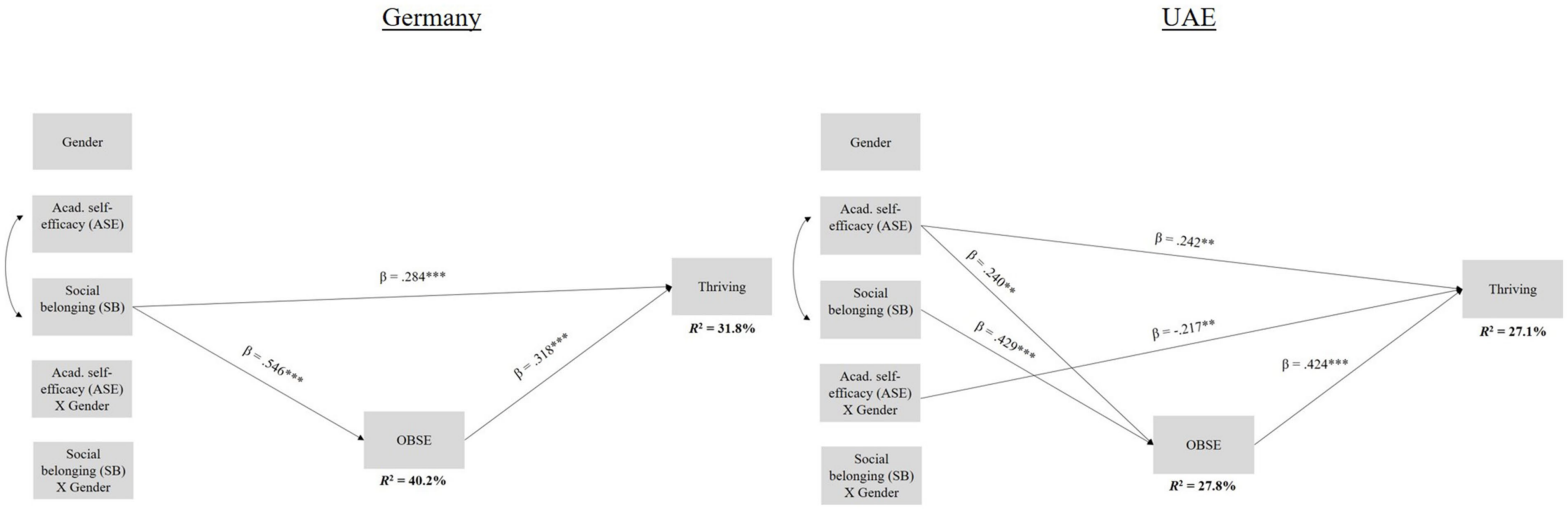
*N* = 489. Germany: *n* = 259; UAE: *n* = 230. Values were estimated using Mplus and full information likelihood estimation (FIML). 0 = Germany, 1 = UAE; *p < 0.05; **p ≤ 0.01; ***p ≤ 0.001. Indirect effects: Germany: partial mediation for SB (*β* = 0.173 *p* ≤ 0.001, 95% CI = 0.078 to 0.264); UAE: partial mediation for ASE (*β* = 0.102, *p* ≤ 0.01, 95% CI = 0.030 to 0.198), full mediation for SB (*β* = 0.182, *p* ≤ 0.001, 95% CI = 0.093 to 0.284).

With regard to research question 1, that is, whether ASE (individual coping resource) would predict thriving, in Analysis 1, we found ASE to be a positive predictor of thriving in Indonesia (*β* = 0.282, *p* ≤ 0.001; see Figure 1). In Analysis 2, ASE (*β* = 0.242, *p* ≤ 0.01) positively predicted thriving in the UAE (see Figure 2). With respect to research question 2, which focused on SB (social coping resource) as a potential predictor of thriving, in Analysis 1, we found SB to be a positive predictor of thriving in Germany (*β* = 0.286, *p* ≤ 0.001) and in Indonesia (*β* = 0.165, *p* ≤ 0.001; see Figure 1). In Analysis 2, SB again directly contributed to students’ thriving in Germany (*β* = 0.284, *p* ≤ 0.001; see Figure 2). Our third research question was whether OBSE mediated these relationships. In Analysis 1, in Germany, with regard to the relationship between students’ SB (*β* = 0.149, *p* ≤ 0.01, 95% CI = 0.049 to 0.248) and thriving, we found a significant partial mediation with OBSE as a mediator (see Figure 1), confirming our hypothesis. In Indonesia, we found significant partial mediations for both coping resources (ASE: *β* = 0.095, *p* ≤ 0.001, 95% CI = 0.055 to 0.141; SB: *β* = 0.073, *p* ≤ 0.001, 95% CI = 0.041 to 0.108; see Figure 1), again in line with our expectations regarding OBSE as a mediator between SB and thriving. In Analysis 2, with regard to the relationship between students’ SB (*β* = 0.173, *p* ≤ 0.001, 95% CI = 0.078 to 0.264) and thriving, we found a significant partial mediation with OBSE as a mediator in Germany, as expected (see Figure 2). In the UAE, we found a partial mediation for ASE (*β* = 0.102, *p* ≤ 0.01, 95% CI = 0.030 to 0.198) and a full mediation for SB (*β* = 0.182, *p* ≤ 0.001, 95% CI = 0.093 to 0.284; see Figure 2), again, in line with our expectations with regard to OBSE as a mediator between SB and thriving. Regarding the potential role of gender in these relationships (research question 4), in Analysis 1, we found no moderation effect of gender, in line with our expectations with regard to ASE as a potential direct predictor of thriving. In Analysis 2, against our hypothesis, the interaction between gender and ASE (*β* = −0.217, *p* ≤ 0.01) negatively predicted thriving in the UAE (see Figure 2). Thus, female students with higher values of ASE were more likely to thrive than their male fellow students; post-hoc simple slopes analyses confirmed this finding. In Analysis 1, the overall model explained a total of 28.3% of the variance in thriving in Germany and a total of 32.2% in Indonesia. In Analysis 2, the overall model explained a total of 31.8% of the variance in thriving in Germany and a total of 27.1% in the UAE.

Our analyses to examine whether the regression weights differed significantly for both countries, showed that, in Analysis 1, chi-square statistics were significant, *χ*^2^ (16) = 75.452, *p* ≤ 0.001, which indicates differences in parameter estimates between Germany and Indonesia. In Analysis 2, chi-square statistics were also significant, *χ*^2^ (16) = 59.910, *p* ≤ 0.001, which also confirms differences in parameter estimates between Germany and the UAE.

## 5 Discussion

Thriving is a specific experience of growth, characterized by a positive sense of vitality and learning, and can be considered an important adaptive response of university students to study-related stressors. To our knowledge, this is the first cross-cultural study that has examined university students’ thriving and its predictors (i.e., coping resources: academic self-efficacy, ASE; social belonging, SB) in one country typically assigned to individualistic cultures (i.e., Germany, Analysis 1 and Analysis 2), and compared it to two countries assigned to collectivistic cultures (i.e., Indonesia, Analysis 1; UAE, Analysis 2), while considering gender as a potential moderator in these relationships. To better understand the psychological mechanism underlying the impact of coping resources on experiences of thriving, we examined to what extent students’ feelings of being seen and valued by their peers and instructors, i.e., their organization-based self-esteem (OBSE), served as a linchpin in transforming their individual and social coping resources into thriving. To that end, we analyzed the role of OBSE as a mediator between students’ ASE and SB on the one hand and students’ thriving on the other, complementing research on OBSE which has been primarily conducted in work and organizational contexts (for an exception in higher education, e.g., Cameron, 2020).

### 5.1 Transforming coping resources into thriving: the decisive mediating role of students’ OBSE in Germany, Indonesia and the UAE

The key result of our study is that, converging with previous findings in individualistic and collectivistic non-academic settings (e.g., Gardner et al., 2018; Kim and Beehr, 2020, 2022; Yang et al., 2018), we found significant indirect effects with OBSE as a mediator in all three countries—irrespective of students’ gender. Although the cross-sectional study design does not allow inferences about causality, our results do indicate that university students’ feelings of being valued, seen and important is a relevant mechanism for transforming the beneficial effects of their coping resources and, thus, promoting thriving under challenging conditions.

The most central result with regard to these mediations is that OBSE served as a mediator between a *social* coping resource (i.e., SB) and female as well as male students’ thriving in all three countries. When facing challenging study-related circumstances, feelings of being a part of their respective study program may have converged with experiences of feeling valued by peers and instructors (see Bowling et al., 2010). This form of social validation, in turn, contributed to students’ sense of vitality and learning (e.g., Feeney and Collins, 2015; Kim and Beehr, 2020; Zhou et al., 2023b). Our findings are in line with research from both individualistic and collectivistic cultures that has found OBSE to serve as a mediator between social factors such as feedback and support, and positive work-related outcomes such as thriving and satisfaction (e.g., Kim and Beehr, 2020; Yang et al., 2018). Consistent across Germany, Indonesia and the UAE, our results once again underscore the universal need to belong (Allen et al., 2021; Baumeister and Leary, 1995) and the importance of actually feeling included at university as a cross-culturally relevant social resource for coping with challenges (e.g., Cortina et al., 2017; Li et al., 2025; Walton and Cohen, 2011). The results further point to the crucial role of a context-specific sub-dimension of students’ self-esteem, that is, their OBSE. Self-esteem itself is contingent on one’s perception of the quality of their relationships with significant others (Leary, 2005; Leary and Baumeister, 2000; see also Perinelli et al., 2022). Resulting from the interactions of a person with others in a given context (Allen et al., 2021, 2022; Goodenow, 1993), students’ SB can be considered an indicator of students’ perceived quality of their interpersonal relationships with peers and instructors in their study program (e.g., Shook and Clay, 2012; Walton and Cohen, 2007; see also Li et al., 2025). The original measure on which we based our measure of SB was developed to assess the perceived inclusion of others in one’s self through overlapping selves (Aron et al., 1992). According to Aron et al. (1992), individuals feel close and relate to others when they include various aspects of them—such as characteristics, perspectives, and resources—into their own self. Transferring this idea to higher education in different cultural contexts, it can be assumed that students feel included when they experience characteristics or elements of their academic environment (including peers and instructors) like integrative parts of their own self and identity. Due to these feelings of belonging students may recognize and utilize social coping resources effectively, and, for instance, could be more likely to seek help when facing challenges (see Zander and Höhne, 2021). In line with that, the experience of SB is often positively related to the access of social support from family and friends, as well as from peers and instructors in higher education (e.g., Du et al., 2023; Mtshweni, 2024; Walton and Cohen, 2007; for schools, see Chiu et al., 2016). A stronger sense of SB, in turn, can make students more sensitive to cues that affirm their value and importance within their study program, thus increasing their OBSE. This, in turn, can promote students’ vitality and learning under challenging circumstances by leveraging their coping resources. This experience of thriving has, in turn, the potential to promote students’ learning-relevant outcomes such as their performance and career intentions (e.g., Ozcan et al., 2023; for work contexts, see Christensen-Salem et al., 2021).

Despite this potential universality, considering the lack of measurement invariance of SB in our study, it should be noted that it is not clear which unit of reflection students used when estimating their SB. It may well be that students in Germany used an individual unit of reflection, and those in Indonesia and the UAE used a social unit of reflection (Markus and Kitayama, 1991; see also Baumeister and Leary, 1995).

### 5.2 The cross-cultural importance of coping resources promoting female and male university students’ thriving

With regard to the direct effects of coping resources on students’ thriving, confirming prior research (e.g., Yang et al., 2023), we found ASE to directly catalyze thriving in Indonesia and the UAE. In general, this is in line with research showing that individuals’ positive beliefs about their personal future can enable them to thrive under challenging circumstances (Carver, 1998; Park, 1998; see also Brown et al., 2017). However, ASE did not predict students’ thriving in Germany. Although our study does not permit causal explanations, we would like to propose several interpretations for these differential results. These interpretations relate primarily to the academic cultures specific to each country (e.g., Hofstede, 1986; Irnidayanti and Fadhilah, 2023; McMinn et al., 2022), but also to Hofstede’s broader cultural dimensions (Hofstede, 1980, 2001, 2011), which may seem partially contradictory at first glance.

In Germany, it may appear surprising that ASE was not positively associated with thriving, particularly given evidence that self-enhancement is often strongly pronounced in individualistic cultures (e.g., Falk et al., 2009; Heine and Hamamura, 2007; Salvador et al., 2024). One might have expected that such self-orientation would have made ASE an important factor for students’ experiences of vitality and learning. However, this pattern was not observed, potentially due to the learner-centered, egalitarian academic culture prevalent in German higher education (Hofstede, 1986, 2001). In such environments, instructors treat students as equals, and collaborative learning is often emphasized (BMBF, 2020). Consequently, German students may have relied more on interpersonal relationships—reflected in their SB—to experience thriving, rather than on their ASE. By contrast, in high power distance cultures (e.g., Indonesia), instruction in educational institutions is typically instructor-centered and strictly disciplined (e.g., Irnidayanti and Fadhilah, 2023). Thus, in Indonesia, where students are generally expected to prepare their coursework on their own, students may have relied also on their perceived individual performance-related abilities in their studies (i.e., their ASE) to experience thriving. With regard to the UAE, the importance of ASE could be explained by the relatively young development-orientated Emirati private higher education system characterized by modernization, internationally focused study programs and economic diversification goals (e.g., Ashour and Kleimann, 2024). Within this higher education context, students may have especially relied on their individual positive beliefs about their personal future to experience growth (see Brown et al., 2017; see also Al Amimi and Ahmad, 2023)—alongside a relatively young learner-centered academic culture (see Sabah and Du, 2018).

In addition, one further explanation for the importance of ASE in Indonesia and the UAE, but not in Germany could be the statistically lower levels of ASE among students in Indonesia and the UAE in our study, compared to Germany (see Tables 1, 3), which might have had a stronger impact on students’ thriving. Indeed, there is evidence that lower levels of certain factors can exert a more pronounced influence on positive outcomes (e.g., Pavani et al., 2019). This aligns with findings that students in collectivistic cultures often report lower levels of self-efficacy compared to students in individualistic cultures (e.g., Jin et al., 2023). These lower levels of self-efficacy in collectivistic cultures are often explained by self-efficacy being shaped more by group success than by personal accomplishments, aligning with cultural norms that prioritize collective achievement (e.g., Bonneville-Roussy et al., 2019; Kitayama and Uskul, 2011; Oettingen, 1995; Wang et al., 2020).

While the contribution of ASE to students’ thriving was consistent across gender in Indonesia, in the UAE, female students with higher values of ASE were more likely to thrive than their male peers. One explanation for the interaction may be gender inequality in terms of social and economic status in the UAE. Although women surpass men in higher education enrollment (The World Bank, 2024), various (sub-)cultural and familial factors such as gender-role socialization, religion and maternal education create greater barriers for women entering a profession after graduation compared to men (Arab Center Washington DC, 2020). Accordingly, and despite from several legal reforms aiming at strengthening women’s economic participation, the female labor force participation rate in the UAE (57.5% in 2020) remains considerably lower than that of males (92% in 2020; Hamel and Dexter, 2021). Thus, in our study, female students in the UAE coming from different Arab countries, who have made their way into higher education against more traditional gender role prescription may have felt the need to rely more on their self-assessed performance-related abilities, driven by individual effort, to experience thriving within their collectively shaped, yet economically still male-dominated, context (see Aldridge and Rowntree, 2022; McClusky and Allen, 2023). This is particularly interesting in light of research showing that gender inequality is sometimes perceived as less unfair by women in collectivistic cultures compared to women in individualistic cultures (e.g., Kinias and Kim, 2012), and that it has been found to be negatively related to well-being among women from individualistic cultures, but not among those from collectivistic cultures (e.g., Li et al., 2025). Although experiences of thriving via social factors are suggested to be contingent on gender role beliefs, which, in turn, can be shaped by culture, SB was a direct relevant catalyzer for both female and male students’ thriving in Germany and Indonesia. This is in line with research showing that German men assess themselves similarly to German women in terms of orientation toward communal goals (e.g., Obiama et al., 2022), and that in collectivistic cultures emphasizing community and union creation can be generally expected, irrespective of gender (e.g., Steinmetz et al., 2014).

### 5.3 Limitations and future directions

While our study significantly contributes to understanding university students’ thriving in different cultural contexts, several limitations suggest avenues for future research. First, the correlational nature of our data forbids causal interpretations regarding thriving and its predictors. Although, in our surveys, coping resources and OBSE were assessed before students’ thriving, and therefore, it seems plausible that thriving is indeed an outcome of resources and OBSE, longitudinal designs should address this limitation in future research. Because thriving can vary over time and across contexts, with changes in intraindividual levels in small or medium periods of time, particularly during substantial changes in a person’s work life (e.g., Bensemmane et al., 2018; Brown et al., 2021; Kleine et al., 2023), future research could also benefit from designs modeling longitudinal intraindividual change. Second, research should also examine the potential reciprocal relationships between OBSE and SB. In contrast to our findings, for example, a study has shown that self-esteem predicted school belonging of Mexican-origin students, but not vice versa (Hernández et al., 2017). This also seems important, given that OBSE and SB are commonly considered distinct factors, yet share some conceptual overlap (e.g., Ma et al., 2025; see also Allen et al., 2021; Bowling et al., 2010).

Third, while our analyses supported (partial) scalar invariance for OBSE and thriving, testing measurement invariance was not possible for our two-item measure of ASE and our one-item measure of SB. Due to the reference-group effect, which can arise when participants from different cultures use different reference groups (often their own cultural group) while responding to self-report scales (see Heine et al., 2002; see also Lacko et al., 2022), the specific items of ASE and SB in our study—originating from WEIRD contexts (Jerusalem and Schwarzer, 1986; Kreutzmann et al., 2018)—may not have had the same psychological meaning across countries (e.g., Jin et al., 2023; see also Van de Vijver and Poortinga, 2002). Furthermore, we are aware of the generally limited reliability and construct validity of ASE and SB in our study, as such short scales may fail to capture the full complexity of psychological constructs (e.g., Ziegler et al., 2014) and do not provide sufficient control over measurement errors (e.g., Davidov et al., 2018). However, with regard to ASE, we were only able to demonstrate satisfactory internal consistency of the scale in all three countries using these two items, consistent with previous research highlighting the challenges associated with internal consistency of measures in cross-cultural studies (e.g., Mushquash and Bova, 2007). Despite this issue, as already noted, we deemed it important to investigate a key individual predictor of students’ thriving for which evidence already exists: their self-efficacy (see Brown et al., 2017). Further, to minimize participant fatigue—a common challenge in longer survey studies (e.g., Jeong et al., 2023)—we used a one-item measure for SB adapted from an original scale (Aron et al., 1992) that was recommended as a widely useful measure when participants’ time is scarce (as was the case in our surveys) or to achieve comparability between diverse populations and situations. Still, future research should continue to develop novel techniques for examining measurement invariance of one-item measures across cultures (e.g., Raudenská, 2023).

Fourth, our study is limited to three specific universities with unique characteristics and, thus, generalizations about the examined countries should be made carefully. In addition to the already mentioned, somewhat contradictory aspects of the cultural dimensions proposed by Hofstede (1980, 2001, 2011), i.e., individualism and power distance, which are related to different academic cultures (Hofstede, 1986, 2001), the national higher education systems in which students in our study pursued their degrees differ in terms of the proportion of state versus private higher education institutions, unequal conditions for access (e.g., related to affordability, rurality, or gender), and the proximity to religion and the state, among other factors (e.g., Ashour, 2020; KMK, 2023; Welch and Aziz, 2022). While the university in Germany in our study is a state institution with no links to religion, the state university in Indonesia incorporates Islamic knowledge into its vision. The university in the UAE is a small private for-profit university. Future studies on thriving should therefore differentiate between different types of higher education institutions and include specific information on academic cultures, including teaching forms and classroom arrangements. Such differentiation seems especially relevant given the lack of measurement invariance between Indonesia and the UAE in our study—despite their comparable scores on individualism and power distance (Hofstede, 1980, 2001, 2011). This suggests that broad cultural indicators may not fully determine how psychological constructs like OBSE and thriving are understood and experienced in different cultural educational contexts (see Fischer et al., 2025). The lack of measurement invariance not only reflects statistical non-equivalence, but also indicates different semantic (e.g., cultural, academic, institutional) meanings across groups, as measurement invariance depends on factors such as item interpretation (see Davidov et al., 2018; Fischer et al., 2025). Thus, the lack of measurement invariance between Indonesia and the UAE may reflect differences in institutional framings of OBSE, as well as vitality and learning, shaped by the heterogeneity of teaching practices—such as problem-based learning or group discussions—resulting from recent educational reforms in the UAE (e.g., McMinn et al., 2022), compared to a more traditional, homogeneous academic culture in Indonesia (e.g., Irnidayanti and Fadhilah, 2023). The lack of measurement invariance between these two countries may have also been related to the heterogeneous composition of the UAE sample, which included students from Lebanon, Egypt, Jordan, and Syria. This within-sample diversity, in terms of cultural and educational backgrounds, may have been associated with different (prior) educational experiences of OBSE, as well as vitality and learning, compared to the more homogeneous sample in Indonesia. That said, it is important to note that Lebanon, Egypt, Jordan, and Syria share meaningful cultural and educational commonalities with the UAE, as evidenced by their similar scores on individualism and power distance (Hofstede, 1980, 2001, 2011), as well as by their comparable trends toward Western academic culture, particularly in terms of learner-centered curricular reforms, English-medium instruction, and internationalization efforts, including the diversification of the student body (e.g., Abdel Latif and Alhamad, 2023; Azoury and Habchi, 2023; Marchesini, 2021). The finding of measurement invariance between Germany and each of the other two countries, which may seem counterintuitive given their more pronounced cultural differences based on Hofstede’s dimensions (Hofstede, 1980, 2001, 2011), suggests that students in both comparisons may have interpreted the items in a statistically comparable way (see Davidov et al., 2018; Fischer et al., 2025).

With regard to our different samples, it should also be noted that there was a (significantly) higher proportion of Bachelor students in our samples from Indonesia and the UAE, compared to Germany. These differences in students’ academic levels may have been related to different experiences of individual and social coping resources, OBSE, and thriving. For instance, levels of positive learning-related outcomes such as engagement, which is conceptually close to thriving, can vary between undergraduate and postgraduate students (e.g., Kobicheva, 2022). Future research on thriving in higher education should therefore control for academic level or include it as a moderating variable to better understand its potential impact on these constructs.

Fifth, the distinction individualism–collectivism as dimensions of national culture does not capture person-level individualistic and collectivistic tendencies of our participants (i.e., idiocentrism; allocentrism), within-country variation, and cultural diversity (e.g., Eom et al., 2016; Fatehi et al., 2020; Green et al., 2005; Klein et al., 2024; Oyserman et al., 2002; Suharnomo and Syahruramdhan, 2018; Triandis et al., 1990; Varnum et al., 2010; Voronov and Singer, 2002). We therefore may have overestimated the importance of the cultural context as a whole. Lastly, because affective and cognitive dimensions of psychological experiences are closely intertwined (e.g., Conte et al., 2023; Eagly and Chaiken, 1993; Levine, 2022; Nejati, 2025; see also Falon et al., 2023), and following prior research (Spreitzer et al., 2005), we conceptualized thriving as a joint experience of vitality (affective) and learning (cognitive). However, because both dimensions are strongly related to culture (for emotional experiences, i.e., vitality, see Fang et al., 2018; Frenzel et al., 2007; Grossmann et al., 2016; Kitayama et al., 2006; Laukka and Elfenbein, 2021; Miyamoto et al., 2010; for learning see Lam and Zhou, 2022; Rosenthal and Feldman, 1991), future validation studies should examine the two dimensions of thriving separately to account for different psychological meanings of vitality and learning (for a validation in a Turkish higher education context, see Ozcan et al. (2023); for a cross-cultural validation on a different conceptualization of thriving, see Wiese et al., 2018).

## 6 Conclusion

The present results shed light on understanding a positive university students’ response to study-related stressors: the experience of vitality, learning and growth. Strengthening students’ individual and social coping resources and enhancing their feelings of being valued and important during their studies in order to promote their thriving remain important goals of higher education institutions across diverse cultural contexts.

## Data Availability

The raw data supporting the conclusions of this article will be made available by the authors, without undue reservation.

## Footnotes

^1^According to social role theory (Eagly, 1987; Eagly and Wood, 2012), gender role beliefs represent people’s stereotypic perceptions of women’s and men’s social roles in their respective society and are often generalized ideas about behavioral differences and similarities between women and men.
