# Supplementary material for: Feeling seen matters: how organization-based self-esteem mediates the relationship between university students’ coping resources and thriving in Germany, Indonesia, and the United Arab Emirates
Source: Front Psychol. 2025 Sep 10;16:1527121. doi: 10.3389/fpsyg.2025.1527121 (PMC12457349; doi:10.3389/fpsyg.2025.1527121)
Supplement: Supplementary file 1 [file Data_Sheet_1.docx]

Supplementary Material

# Preliminary Analyses

Measurement invariance across countries was tested for thriving and OBSE within the framework of multiple-group confirmatory factor analyses (MGCFA). In both analyses, in addition to the chi-square difference test (Δχ²; Anderson and Gerbing, 1988), we evaluated measurement invariance by examining changes in model fit indices, following the guidelines by Chen (2007). We assumed measurement invariance when models differed by no more than 0.010 in CFI, supplemented by 0.015 in RMSEA or 0.030 in SRMR (ibid.). These cutoffs were applied when comparing increasingly constrained models – that is, metric vs. configural and scalar vs. metric (see also Goretzko et al., 2023).

## Analysis 1 (Germany and Indonesia)

The configural invariance model for thriving showed excellent fit (χ2 = 0.000; df = 0; *p* ≤ 0.001; CFI = 1.000; RMSEA = 0.000 (90% CI = 0.000/0.000); SRMR = 0.000). The fit of the metric invariance model was also good (χ2 = 5.357; df = 2; *p* = 0.069; CFI = 0.994; RMSEA = 0.062 (90% CI = 0.000/0.129); SRMR = 0.047), and imposing constraints on the factor loadings did not diminish model fit (Δχ2 = 5.357; df = 2; *p* = 0.069). Although changes in RMSEA and SRMR slightly exceeded the recommended cutoffs by Chen (2007), we considered metric invariance for thriving to be sufficiently supported based on the non-significant chi-square test. This decision is consistent with the notion that such cutoffs should be applied more flexibly rather than rigidly – particularly in models with low degrees of freedom and in cross-cultural comparisons, where slight changes in fit indices may reflect sampling variation rather than true non-invariance (e.g., Barrett, 2007; Byrne and van de Vijver, 2010; Davidov et al, 2018; Fischer et al., 2025; Groskurth et al., 2023; Kenny et al., 2015; Milfont and Fischer, 2010; Putnick and Bornstein, 2017; Zhao et al., 2024). Moreover, the CFI and its changes – which, in this case, remained within the recommended cutoff (Chen, 2007) – has been proposed as one of the most effective indicators of true non-invariance (e.g., Meade et al., 2008; see also Neufeld et al., 2023). After adding constraints on item intercepts across countries, model fit was still good (χ2 = 6.172; df = 4; *p* = 0.187; CFI = 0.996; RMSEA = 0.035 (90% CI = 0.000/0.087); SRMR = 0.052), and showed no reduction in model fit compared to the metric one (Δχ2 = 0.815; df = 2; *p* = 0.665). Thus, scalar invariance was supported.

For organization-based self-esteem (OBSE), the fits of the configural invariance model (χ2 = 0.000; df = 0; *p* ≤ 0.001; CFI = 1.000; RMSEA = 0.000 (90% CI = 0.000/0.000); SRMR = 0.000) and of the metric invariance model (χ2 = 0.688; df = 2; *p* = 0.709; CFI = 1.000; RMSEA = 0.000 (90% CI = 0.000/0.069); SRMR = 0.015) were good. The metric invariance model showed no significant reduction compared to the configural one (Δχ2 = 0.688; df = 2; *p* = 0.709), supporting metric invariance. After adding constraints on item intercepts across countries, model fit was still good (χ2 = 3.475; df = 4; *p* = 0.482; CFI = 1.000; RMSEA = 0.000 (90% CI = 0.000/0.068); SRMR = 0.012), and showed no reduction in model fit compared to the metric one (Δχ2 = 3.00; df = 2; *p* = 0.223), thereby supporting scalar invariance.

## Analysis 1 (Germany and UAE)

The configural invariance model for thriving was a good fit to the data (χ2 = 0.000; df = 0; *p* ≤ 0.001; CFI = 1.000; RMSEA = 0.000 (90% CI = 0.000/0.000); SRMR = 0.000). The fit of the metric invariance model was also good (χ2 = 1.067; df = 2; *p* = 0.587; CFI = 1.000; RMSEA = 0.000 (90% CI = 0.000 /0.114); SRMR = 0.023), and imposing constraints on the factor loadings did not diminish model fit (Δχ2 = 1.067; df = 2; *p* = 0.587). Thus, metric invariance was supported. After adding constraints on the intercepts of item 1 and 4, model fit was still good (χ2 = 3.998; df = 4; *p* = 0.406; CFI = 1.000; RMSEA = 0.000 (90% CI = 0.000 /0.105); SRMR = 0.044), and showed no reduction in model fit compared to the metric one (Δχ2 = 3.115; df = 2; *p* = 0.211). Thus, partial scalar invariance was supported.

For organization-based self-esteem (OBSE), the fits of the configural invariance model (χ2 = 0.000; df = 0; *p* ≤ 0.001; CFI = 1.000; RMSEA = 0.000 (90% CI = 0.000/0.000); SRMR = 0.000) and of the metric invariance model (χ2 = 0.104; df = 2; *p* = 0.949; CFI = 1.000; RMSEA = 0.000 (90% CI = .000/.008); SRMR = 0.008) were good. The metric invariance model showed no significant reduction compared to the configural one (Δχ2 = 0.104; df = 2; *p* = 0.949), supporting metric invariance. After adding constraints on item intercepts across countries, model fit was still good (χ2 = 5.006; df = 4; *p* = 0.287; CFI = 0.996; RMSEA = 0.035 (90% CI = 0.000/0.114); SRMR = 0.027), and showed no reduction in model fit compared to the metric one (Δχ2 = 5.169; df = 2; *p* = 0.075). Thus, scalar invariance was supported.

# Additional Analyses

Table 1

*Sociodemographic characteristics of participants separated by country*

| **Characteristic** | **Germany** | | **Indonesia** | | **UAE** | |
| --- | --- | --- | --- | --- | --- | --- |
|  | *n* | *%* | *n* | *%* | *n* | *%* |
| **Gender** |  | |  | |  | |
| Female | 132 | 51% | 508 | 60.6% | 152 | 66.1% |
| Male | 46 | 17.8% | 126 | 15% | 74 | 32.2% |
| **Degree** |  |  |  |  |  |  |
| Bachelor | 90 | 34.8% | 564 | 67.2% | 219 | 95.2% |
| Master | 96 | 37.1% | 68 | 8.1% | 3 | 1.3% |
| **Teacher Training** |  |  |  |  |  |  |
| Yes | 120 | 46.3% | 595 | 70.9% | 28 | 12.2% |
| No | 64 | 24.7% | 36 | 4.3% | 190 | 82.6% |
| **Academic Discipline** |  |  |  |  |  |  |
| Humanities and Social Sciences | 259 | 100% | 583 | 69.4% | 101 | 43.9% |
| Business and Law | 0 | 0% | 30 | 3.6% | 57 | 24.8% |
| STEM | 0 | 0% | 12 | 1.4% | 51 | 22.2% |

*Note.* Germany: *n* = 259; Indonesia: *n* = 839; UAE: *n* = 230. Participants in Germany were on average 24.49 (*SD* = 4.69), in Indonesia, 21.15 (*SD* = 4.01), and in the UAE, 19.77 (*SD* = 1.78) years old. In Germany, 81 (31.2%), in Indonesia, 205 (24.4%), and in the UAE, 4 (1.7%) of the students did not indicate their gender. In Germany, 73 (28.1%), in Indonesia, 207 (24.7%), and in the UAE, 8 (3.5%) of the students did not indicate their degree. In Germany, 75 (29%), in Indonesia, 208 (24.8%), and in the UAE, 12 (5.2%) of the students did not indicate if they were studying as part of a teacher training program. In Indonesia, 214 (25.6%) of the students did not indicate their academic discipline. In the UAE, 21 (9.1%) of the students did not indicate their academic discipline. The category “Humanities and Social Sciences” in Germany primarily included subjects in the fields of educational sciences, teacher training programs, psychology, and languages. The category “Humanities and Social Sciences” in Indonesia primarily included subjects in the fields of teacher training programs and early childhood education. The category “Humanities and Social Sciences” in the UAE primarily included subjects in the fields of psychology, visual communication, public speaking, and languages.

# References

Anderson, J. C., and Gerbing, D. W. (1988). Structural equation modeling in practice: A review and

recommended two-step approach. *Psychol. Bull.* 103, 411–423. doi: 10.1037/0033-2909.103.3.411

Barrett, P. (2007). Structural equation modelling: Adjudging model fit. *Pers. Individ. Differ*. 42, 815–

824. doi: 10.1016/j.paid.2006.09.018

Byrne, B. M., and van de Vijver, F. J. R. (2010). Testing for measurement and structural equivalence

in large-scale cross-cultural studies: Addressing the issue of nonequivalence. *Int. J. Test*. 10, 107–132. doi: 10.1080/15305051003637306

Chen, F. F. (2007). Sensitivity of goodness of fit indices to lack of measurement invariance. *Struct.*

*Equ. Model*. 14, 464–504. doi: 10.1080/10705510701301834

Davidov, E., Muthén, B., and Schmidt, P. (2018). Measurement invariance in cross-national studies:

Challenging traditional approaches and evaluating new ones. *Sociol. Methods Res*. 47, 631–636. doi: 10.1177/0049124118789708

Fischer, R., Karl, J. A., Luczak-Roesch, M., and Hartle, L. (2025). Why we need to rethink

measurement invariance: The role of measurement invariance for cross-cultural research. *Cross-Cult. Res*. 10693971241312459. doi: 10.1177/10693971241312459

Goretzko, D., Siemund, K., and Sterner, P. (2024). Evaluating model fit of measurement models in

confirmatory factor analysis. *Educ. Psychol. Meas*. 84, 499–524. doi: 10.1177/00131644231163813

Groskurth, K., Bluemke, M., and Lechner, C. M. (2024). Why we need to abandon fixed cutoffs for

goodness-of-fit indices: An extensive simulation and possible solutions. *Behav. Res. Methods* 56, 3891–3914. doi: 10.3758/s13428-023-02193-3

Kenny, D. A., Kaniskan, B., and McCoach, D. B. (2015). The performance of RMSEA in models with

small degrees of freedom. *Sociol. Methods Res*. 44, 486–507. doi: 10.1177/0049124114543236

Meade, A. W., Johnson, E. C., and Braddy, P. W. (2008). Power and sensitivity of alternative fit

indices in tests of measurement invariance. *J. Appl. Psychol*. 93, 568–592. doi: 10.1037/0021-9010.93.3.568

Milfont, T. L., and Fischer, R. (2010). Testing measurement invariance across groups: Applications in

cross-cultural research. *Int. J. Psychol. Res*. 3, 111–130. doi: 10.21500/20112084.857

Putnick, D. L., and Bornstein, M. H. (2016). Measurement invariance conventions and reporting: The

state of the art and future directions for psychological research. *Dev. Rev*. 41, 71–90. doi: 10.1016/j.dr.2016.06.004

Schermelleh-Engel, K., Moosbrugger, H., and Müller, H. (2003). Evaluating the fit of structural

equation models: Tests of significance and descriptive goodness-of-fit measures. *Methods Psychol. Res*. 8, 23–74. doi: 10.23668/psycharchives.12784

Zhao, Y., Summers, R., Gathara, D., and English, M. (2024). Conducting cross-cultural, multi-lingual

or multi-country scale development and validation in health care research: A 10-step framework based on a scoping review. *J. Glob. Health* 14, 04151. doi: 10.7189/jogh.14.04151
